# Therapeutic relationship nurse–patient in hemodialysis therapy

**DOI:** 10.1111/nuf.12590

**Published:** 2021-05-05

**Authors:** Marta Hreńczuk

**Affiliations:** ^1^ Department of Surgical, Transplantation Nursing, and Extracorporeal Treatment, Faculty of Health Sciences Medical University of Warsaw Warsaw Poland

**Keywords:** hemodialysis, quality of care, therapeutic relationship

## Abstract

**Introduction:**

Building a therapeutic relationship aims to achieve therapeutic goals.

The aim of the study was to determine the importance of the therapeutic relationship between nurse and patient in hemodialysis therapy.

**Material and Methods:**

The study included 77 patients with end‐stage kidney disease (ESKD) and on long‐term hemodialysis in one of Warsaw's dialysis centers. The diagnostic survey method was used with the questionnaire technique. A survey questionnaire developed by the author was used as the tool for the study. The analysis was carried out using StatSoft Statistica 13.1 PL statistical package and Microsoft Office.

**Results:**

According to the respondents, the main elements of a therapeutic relationship are empathy (82%), mutual trust (68%), and respect (60%). 86% of patients believed that the nurse–patient relationship was important in renal replacement therapy, significantly less frequently with reference to patients aged 70 years and older (*p* < .05). Patients positively evaluated the nurse‐patient therapeutic relationship, indicating that it influences the sense of security, helps in coping with difficult moments, and increases the quality of medical care.

**Conclusions:**

The establishment of a therapeutic relationship with the patient should be the basic course of action of every nurse.

## 1 INTRODUCTION

Therapeutic relationship is an increasingly common concept in the medical literature. In the broadest sense, it refers to any nonspecific psychological interaction related to treatment. Mutual cooperation with the patient is based, to a large extent, on the formation of an appropriate therapeutic relationship, that is, on a positive emotional relationship with the patient, who follows the tasks that aim at achieving therapeutic goals.^1^, ^2^, ^3^

One of the important needs of a man, especially the sick one, is the need to feel safe. Many patients do not have even elementary knowledge in the field of medicine. A helpless, dependent, and fearful patient who feels at risk usually seeks information, explanations, and words that will bring reassurance and understanding. For the relationship to be effective, it is necessary for the contact between the patient and the nurse to be good.^4^, ^5^ An irreplaceable element of a good and fruitful therapeutic relationship is empathy. Empathy is the ability to step into another person's shoes and to identify with that person's emotional state, behavior, attitude, and feelings.^6^ An empathetic person is one who tries to understand and accept another person's point of view without making critical judgments and showing hostility. Empathy protects against the duplication of patterns and routine in contacts with the sick. In the context of the clinical situation, however, it is often equated with the ability to be fully with the other person. Being so fully present also means being able to see when to be active and when to be an observer, when to speak honestly and when to refrain from commenting. It can be stated that empathy is not only a moral and philosophical issue but one of the most important competencies of a modern nurse. Without empathy there is no trust, without trust, there is no sincere and good therapeutic nurse‐patient relationship, which is helpful in the treatment process.

A chronic disease is undoubtedly one source of stress that can be experienced in human life. It is a difficult and complex situation in many aspects—cognitive, emotional, social, and existential. It requires the patient, and often his immediate environment, to cope with difficulties resulting from the disease itself as well as from therapy.^7^ A chronic kidney disease is one such type of disease, which requires renal replacement therapy in its final stage. This treatment includes dialysis (peritoneal dialysis, hemodialysis) or kidney transplantation. The first mentioned therapy is characterized by repeated procedures until transplantation if there are no contraindications, or until the end of life. Estimates are that 2 million people worldwide suffer from kidney failure, and the number of patients diagnosed with the disease continues to increase at a rate of 5‐7% per year.^8^ The population of patients receiving dialysis continues to grow rapidly. Worldwide, approximately 89% of patients on dialysis receive hemodialysis.^9^ Hemodialysis is the dominant therapy in the world. Therefore, the following research question was posed: what is the perception of the therapeutic relationship by hemodialysis patients?

The aim of the study was to determine the importance of the therapeutic relationship between the nurse and the patient in the treatment of patients undergoing chronic treatment with hemodialysis.

## 2 MATERIAL AND METHODS

The study was conducted in the period from November 2019 to January 2020. It included 89 people who were chronically hemodialysed during this period at the dialysis center of the University Clinical Center of Baby Jesus Clinical Hospital located at 4 Lindleya street. The study was voluntary and anonymous, and 77 people completed the survey. The percentage of people who agreed to complete the survey is 86.5% of all dialysis station patients. The research method used in the study was a diagnostic survey with the use of a proprietary questionnaire developed exclusively for this study. The study conforms to recognized standards Declaration of Helsinki. The study was acknowledged by the Bioethics Committee of the Medical University of Warsaw (decision: AKBE/307/2019).

The results are presented with regard to qualitative data by cardinality and percentage, whereas in relation to quantitative data by mean, standard deviation, median, as well as minimum and maximum values. To check whether there are statistically significant dependencies among the variables, an analysis was performed using the nonparametric Pearson's Chi‐square test for qualitative data. The quantitative data distribution was examined using the Shapiro–Wilk test. After determining the distribution (inconsistent with normal), the Mann–Whitney *U* test (UMW, Z) was used to compare the two groups. The Spearman (R) correlations were applied. It was assumed that "*p*" with a value lower than 0.05 indicated the presence of a statistically significant relationship. The analysis was carried out using StatSoft Statistica 13.1 PL statistical package and Microsoft Office.

### 2.1 The characteristics of the study group

In the study group, women constituted 53.2%, and men 46.8%. The youngest patient was 21 years old and the oldest was 88 years of age. The average age of the respondents was 51.8 years old. The biggest group of respondents were people aged 40–50. Nearly half of the respondents (45.5%) had a university degree. Most of the respondents lived in cities (96.1%). The average duration of hemodialysis treatment was 38.5 months. Most patients were to hemodialysis in a period shorter than 50 months (over 4 years). The subjects were divided into groups on the basis of the length of renal replacement treatment. 2/3 of the patients were treated with hemodialysis for a period from one to 5 years, the smallest number of patients for a period of over 5 years. Most of the respondents (96.1%) accepted the therapy they were participating in. The characteristics of the study group are shown in Table 1.

**Table 1:** The characteristics of the study group

Data		*n*	%
Sex	Women	41	53.2
	Men	36	46.8
Age	<40 years old	20	26.0
	40–49 years old	18	23.4
	50–59 years old	16	20.8
	60–69 years old	12	15.6
	≥70 years old	11	14.3
Education	Basic vocational	17	22.1
	Secondary	25	32.5
	Higher	35	45.5
Place of residence	City	74	96.1
	Village	3	3.9
Duration of hemodialysis	<1 year	18	23.4
	1–2 years	24	31.2
	3–5 years	25	32.5
	>5 years	10	13.0
Acceptance of therapy	Yes	74	96.1
	No	3	3.9

## 3 RESULTS

### 3.1 The concept of the therapeutic relationship and its elements

Most of the surveyed patients (75.3%) understood the concept of a therapeutic relationship well, indicating that it is the expression and mutual exchange of feelings and attitudes, the establishment of a special bond between the patient and the medical staff, which is to serve the treatment process. Less frequently, the respondents reduced this concept to a casual conversation (19.5%) or a purely friendly relation (5.2%). The higher the level of education of the respondents, the statistically more frequently they indicated the correct concept of the therapeutic relationship (vocational: 52.9%, secondary: 80%, higher: 82.9%), *p* < .02.

The respondents listed the elements of the therapeutic relationship—the highest element on the list was empathy (81.8%), then mutual trust (67.5%), and respect (59.7%). Less often, it was believed that the therapeutic relationship consists of the openness of both parties (55.8%), joint verbal contact (54.5%), cordiality (46.8%), nonverbal contact (44.2%), and the sense of security (44.2%), while the least common elements were understanding (39%) and acceptance (33.8%). Empathy as an element of the therapeutic relationship was significantly more often emphasized by men than women (91.7% vs. 73.2%, *p* < .04), people with higher education (vocational: 58.8%, secondary: 84%, higher: 91.4%, *p* < .02), as well as those respondents who did not accept renal replacement therapy in comparison to those that did (100% vs 36.5%, *p* < .03). Understanding was statistically significantly more frequently an important element of the therapeutic relationship for village residents than city residents (100% vs. 36.5%, *p* < .03). Respect as an element of a therapeutic relationship was of particular importance for the respondents with secondary education in comparison to others (76% vs. others: 35.3%–60%), *p* < .03.

### 3.2 The importance of the therapeutic relationship in the opinion of the respondents

The vast majority of patients (85.7%) stated that the therapeutic nurse–patient relationship was important in renal replacement therapy. Four respondents (5.2%) had the opposite opinion, and 9.1% did not express their opinion clearly. Patients aged 70 and more were convinced significantly less often than the younger ones that the therapeutic nurse–patient relationship was important in renal replacement therapy (63.6% vs. younger patients: 3.3%–95%), *p* < .03.

The respondents (*n* = 66) who believed that the therapeutic nurse‐patient relationship was important in therapy mentioned that importance. In most cases, this relationship gave patients a sense of being understood by the other person (33.3%), gave them strength to fight the disease (27.3%), less often helped to restore hope (10.6%), helped them practice the ability to understand their problems by looking at them through the eyes of a different person (9.1%), helped to control helplessness (7.6%), and strengthened the positive aspects of health behaviors (6.1%). Two people mentioned a different meaning—one indicated that it “improves the assessment of the patient in their own eyes”, the other indicated “pleasant conditions of hemodialysis”. Two people replied they “don”t know" (3%).

### 3.3 The respondents' assessment of the influence of the therapeutic relationship on the effects of their therapy

In most cases, the respondents believed that the current therapeutic relationship between the nurse and the patient influences their sense of security (4.5), helps them cope with difficult moments (4.4), and improves the quality of medical care (4.4), but slightly less often they believed that it helps to accept the disease and therapy (4.3) and that it increases the effectiveness of the therapy (4.1). Women significantly more often than men believed that the therapeutic relationship between the nurse and the patient helps to accept the disease and therapy (4.5 vs. 4.1), *p* < .05 (Table 2).

**Table 2:** The respondents' perception of the therapeutic relationships: nurse‐patient, altogether and depending on the variables

The therapeutic nurse–patient relationship:	Total		Sex		Statistics	
	*M*	SD	Women	Men	*Z*	*p*
influences the improvement of the quality of medical care	4.4	0.7	4.5	4.2	1.38	0.168
increases the effectiveness of therapy	4.1	0.7	4.3	3.9	1.82	0.069
influences the sense of security	4.5	0.7	4.7	4.4	1.01	0.312
helps in coping with difficult moments	4.4	0.7	4.6	4.3	1.60	0.110
helps to accept illness and therapy	4.3	0.8	4.5	4.1	1.98	0.048

The therapeutic nurse–patient relationship	Age					Statistics	
	<40 years old	40–49 years old	50–59 years old	60–69 years old	≥70 years old	Spearman's Rho	*p*
influences the improvement of the quality of medical care	4.4	4.3	4.4	4.4	4.2	0.01	0.949
increases the effectiveness of therapy	4.4	3.9	4.2	4.3	4.0	−0.05	0.688
influences the sense of security	4.7	4.6	4.6	4.4	4.4	−0.10	0.370
helps in coping with difficult moments	4.6	4.4	4.6	4.1	4.3	−0.13	0.264
helps to accept illness and therapy	4.5	4.3	4.4	4.1	4.0	−0.18	0.111

The therapeutic nurse–patient relationship	Education			Statistics	
	Vocational	Secondary	Higher	Spearman's Rho	*p*
influences the improvement of the quality of medical care	4.4	4.4	4.3	−0.04	0.729
increases the effectiveness of therapy	4.1	4.3	4.1	−0.05	0.664
influences the sense of security	4.4	4.6	4.5	0.10	0.408
helps in coping with difficult moments	4.5	4.6	4.3	−0.12	0.282
helps to accept the disease and therapy	4.2	4.5	4.1	−0.05	0.661

The therapeutic nurse–patient relationship	Place of residence		Statistics	
	City	Village	*Z*	*p*
influences the improvement of the quality of medical care	4.4	4.7	−0.68	0.494
increases the effectiveness of therapy	4.1	4.3	−0.33	0.742
influences the sense of security	4.5	5.0	−1.09	0.275
helps in coping with difficult moments	4.4	4.7	−0.49	0.626
helps to accept illness and therapy	4.3	4.7	−0.78	0.437

The therapeutic nurse–patient relationship	Duration of hemodialysis				Statistics	
	<1 year	1−2 years	3–5 years	>5 years	Spearman's Rho	*p*
influences the improvement of the quality of medical care	4.4	4.4	4.2	4.5	−0.05	0.650
increases the effectiveness of therapy	4.3	4.3	3.9	4.1	−0.20	0.087
influences the sense of security	4.6	4.8	4.3	4.5	−0.10	0.367
helps in coping with difficult moments	4.4	4.6	4.5	4.0	−0.22	0.058
helps to accept illness and therapy	4.2	4.6	4.2	3.9	−0.17	0.147

The therapeutic nurse–patient relationship	Acceptance of therapy		Statistics	
	Yes	No	*Z*	*p*
influences the improvement of the quality of medical care	4.4	4.7	−0.68	0.494
increases the effectiveness of therapy	4.2	3.7	1.16	0.247
influences the sense of security	4.5	4.7	−0.13	0.895
helps in coping with difficult moments	4.4	4.3	0.45	0.654
helps to accept illness and therapy	4.3	4.0	0.93	0.350

Most of the respondents (87%) believed that the contact of a nurse with a patient in a dialysis center was therapeutic (including ‐ yes: 54.5%, rather yes: 32.5%), two respondents (2.6%) were of the opposite opinion, and 10.4% of the respondents did not express their opinion.

Nearly all of the respondents undergoing dialysis therapy (97.4%) believed that the nurse‐patient relationship was sufficient for them (including ‐ yes: 63.6%, rather yes: 33.8%), two people were of the opposite opinion (2.6%). Women statistically significantly more often than men indicated that the current nurse‐patient relationship was entirely sufficient for them (75.6% vs. 50%), *p* < .04. The shorter the time of the therapy, the statistically significantly more often patients believed that the current nurse–patient relationship was entirely sufficient for them (<1 year: 72.2%, 2–3 years: 70.8%, 3–5 years: 64%, >5 years: 30%), *p* < .01 (Table 3).

**Table 3:** The assessment of satisfaction with the quantity and quality of the nurse‐patient relationship in the dialysis center conducted by the respondents, all together, and depending on the variables

The current nurse–patient relationship is sufficient for you	Total	Sex		Education		
		Women	Men	Vocational	Secondary	Higher
Yes	49	31	18	13	18	18
	63.6%	75.6%	50.0%	76.5%	72.0%	51.4%
Rather yes	26	10	16	4	7	15
	33.8%	24.4%	44.4%	23.5%	28.0%	42.9%
No	2	0	2	0	0	2
	2.6%	0.0%	5.6%	0.0%	0.0%	5.7%
Total	77	41	36	17	25	35
Statistics	–	Chi^2^ = 6.53, *p* = .038		Chi^2^ = 5.56, *p* = .234		

The current nurse–patient relationship is sufficient for you	Duration of hemodialysis (years)				Acceptance of therapy	
	< 1	2–3	3–5	>5	Yes	No
Yes	13	17	16	3	47	2
	72.2%	70.8%	64.0%	30.0%	63.5%	66.7%
Rather yes	5	7	9	5	25	1
	27.8%	29.2%	36.0%	50.0%	33.8%	33.3%
No	0	0	0	2	2	0
	0.0%	0.0%	0.0%	20.0%	2.7%	0.0%
Total	18	24	25	10	74	3
Statistics	Chi^2^ = 16.7, *p* = .010				Chi^2^ = 0.08, *p* = .958	

The current nurse–patient relationship is sufficient for you	Age (in years)					Place of residence	
	<40	40–49	50–59	60–69	≥70	City	Village
Yes	15	11	10	6	7	47	2
	75.0%	61.1%	62.5%	50.0%	63.6%	63.5%	66.7%
Rather yes	5	7	5	6	3	25	1
	25.0%	38.9%	31.3%	50.0%	27.3%	33.8%	33.3%
No	0	0	1	0	1	2	0
	0.0%	0.0%	6.3%	0.0%	9.1%	2.7%	0.0%
Total	20	18	16	12	11	74	3
Statistics	Chi^2^ = 6.38, *p* = .604					Chi^2^ = 0.08, *p* = .958	

A greater involvement of nurses in the therapeutic relationship was expected by 26% of the respondents, including 2.6% yes, and 23.4% rather yes.

All three surveyed village residents rather expected an increase in the involvement of nurses in the therapeutic relationship. City residents significantly less frequently expected an increase in the involvement of nurses in the relationship, *p* < .02. Such action was significantly more often expected by the respondents who had undergone therapy for more than 5 years (yes: 20% vs. the others receiving treatment for a shorter period of time: 0%; rather yes: over 5 years: 40%, others: 12%–33.3%), *p* < .01. All three respondents who were treated with hemodialysis and did not accept this therapy declared that they would rather involve nurses more in the therapeutic relationship, *p* < .02 (Table 4).

**Table 4:** The expectations of the studied patients undergoing hemodialysis regarding the increased involvement of nurses in the therapeutic relationship depending on the variables

You expect greater involvement of the nursing staff in the therapeutic relationship	Age (in years)					Place of residence	
	<40	40–49	50–59	60–69	≥70	City	Village
Yes	0	1	0	0	1	2	0
	0.0%	5,6%	0.0%	0.0%	9.1%	2.7%	0.0%
Rather yes	2	4	7	3	2	15	3
	10.0%	22.2%	43.8%	25.0%	18.2%	20.3%	100%
No	7	7	4	3	3	24	0
	35.0%	38.9%	25.0%	25.0%	27.3%	32.4%	0.0%
Rather not	11	6	5	6	5	33	0
	55.0%	33.3%	31.3%	50.0%	45.5%	44.6%	0.0%
Total	20	18	16	12	11	74	3
Statistics	Chi^2^ = 10.7, *p* = .552					Chi^2^ = 10.2, *p* = .016	

You expect greater involvement of the nursing staff in the therapeutic relationship	Duration of hemodialysis (years)				Acceptance of therapy	
	< 1	2–3	3–5	>5	Yes	No
Yes	0	0	0	2	2	0
	0.0%	0.0%	0.0%	20.0%	2.7%	0.0%
Rather yes	6	5	3	4	15	3
	33.3%	20.8%	12.0%	40.0%	20.3%	100%
No	6	7	8	3	24	0
	33.3%	29.2%	32.0%	30.0%	32.4%	0.0%
Rather not	6	12	14	1	33	0
	33.3%	50.0%	56.0%	10.0%	44.6%	0.0%
Total	18	24	25	10	74	3
Statistics	Chi^2^ = 21.0, *p* = .012				Chi^2^ = 10.2, *p* = .016	

You expect greater involvement of the nursing staff in the therapeutic relationship	Total	Sex		Education		
		Women	Men	Vocational	Secondary	Higher
Yes	2	0	2	0	0	2
	2.6%	0.0%	5.6%	0.0%	0.0%	5.7%
Rather yes	18	7	11	4	2	12
	23.4%	17.1%	30.6%	23.5%	8.0%	34.3%
No	24	14	10	4	9	11
	31.2%	34.2%	27.8%	23.5%	36.0%	31.4%
Rather not	33	20	13	9	14	10
	42.9%	48.8%	36.1%	52.9%	56.0%	28.6%
Total	77	41	36	17	25	35
Statistics	–	Chi^2^ = 4.73, *p* = .192		Chi^2^ = 10.2, *p* = .112		

## 4 DISCUSSION

In 2018, 20418 patients in Poland underwent hemodialysis.^10^ Due to economic reasons, despite numerous arguments in favor, patients in Polish dialysis centers rarely receive psychological assistance. The therapeutic relationship between the nurse and the patient seems to be of significant importance in the group of ESKD patients who undergo repeated hemodialysis treatments. It should be emphasized that the therapeutic relationship, which is most often associated with psychotherapy and psychotherapist, is used in other medical professions, including nursing. Between the nurse and the patient that a specific type of bond is established, which aims to achieve specific behaviors in the patient that strengthen health and support the therapy.^11^

Most of the examined patients correctly understood the concept of a therapeutic relationship. As elements of a therapeutic relationship, the respondents most often mentioned empathy (significantly more often men, people with higher education, and people who do not accept therapy), trust, and respect, while village residents significantly more often indicated understanding. Empathy is the most important component of the therapeutic relationship appreciated by the respondents, which is consistent with the information contained in the literature.^6^, ^12^, ^13^, ^14^, ^15^, ^16^, ^17^ Why are these three values first? By briefly analyzing what a relationship is, one can quote a simple definition that can be found in a dictionary, which reads as follows: “relations are mutual relationships that occur between people or social groups.”^18^ It should be very clearly highlighted that building a therapeutic relationship between a nurse and a patient, with the aim of helping in the treatment process, should be based on empathy, which will allow the sick person to feel understood and accepted. Mutual trust makes it possible to build lasting social relationships. When trust is lacking, people become passive and antisocial, cautious in relations with others, and they stop believing in the effectiveness of any actions.^19^ Mutual trust is necessary for the patient to believe that everything the nurse does for him/her is therapeutic and helpful in treatment. A nurse who trusts a patient does not have to wonder if the patient is following medical recommendations. Such a relationship builds a safety zone in which both parties feel good. On the other hand, the factors described by nurses as significant in the therapeutic relationship in the study by Welch M. include empathy, uniqueness, sense and purpose, as well as appropriate disclosure.^20^

Each of us wants to be respected, especially when we feel that health, which is greatly valued, has been taken away from us by a disease. In nursing practice, it is important to respect difficulties, needs, often pain, various reactions to illness and ailments. Respect should be visible in the holistic approach, represented by the model of care and the philosophy of Primary Nursing, which was established in 1969.^21^ It is important that it refers to all problems, needs, deficits on the part of the patient, often also to the difficulties he or she has in everyday life and the way of experiencing the disease.

The therapeutic relationship in renal replacement therapy is not important to all patients, especially for patients older than 70 years of age. The respondents who considered the therapeutic relation with the nurse important, most often indicated that such a relationship should give a sense of being understood and that it should restore hope. Only a small percentage of patients (6.1%) indicated that it should strengthen positive health behaviors. A relationship, or a bond, as indicated in the literature, may have many aspects, for example, it can be a formal bond maintained by the closeness and availability of a nurse for the patient at any time of the day or night, or a social bond resulting from creating and maintaining an individualized relationship between nurses and patients.^6^, ^22^ It is important to do so, knowing the expectations of patients. However, is what patients expect consistent with what they experience in the therapeutic relationship? To answer this issue, one of the questions asked respondents to evaluate the impact of the therapeutic relationship on the effects of their therapy. In the first place, there were answers that this relationship gives a sense of security, helps in coping with difficult times, helps in accepting one's illness and therapy, and even increases the effectiveness of therapy and the quality of care. In the analyzed group, women significantly more often than men believed that the therapeutic nurse‐patient relationship helps in accepting the disease and the therapy. The nurse‐patient contact is not therapeutic for all respondents, but for the majority (97.4%) it is sufficient. Village residents, patients with dialysis lasting >5 years, and patients who did not accept their therapy expected greater involvement of the nursing staff in the therapeutic relationship. Therefore, with reference to these groups of patients, nurses should show greater commitment to the therapeutic relationship.

Perhaps the lack of involvement of the nursing staff results from their insufficient numbers on the labor market, and thus the lack of time, which is left only for the implementation of instrumental activities. In 2016, there were 5.2 nurses per 1.000 inhabitants in Poland. Unfortunately, this is one of the lowest numbers in the entire European Union, for which the average is 8.4. The largest number of nurses is in Denmark − 16.9 and Finland − 14.3, the smallest in Greece − 3.3.^23^ This state of Polish nursing and workload may be the cause of occupational burnout. Nurses are particularly at risk from this phenomenon. Though their work involves constant changes, saving lives, and the gratitude of patients and their families—the tension, stress, the need to make quick decisions, the responsibility for others, and the constant contact with people, unfortunately, increase the incidence of this disease in staff.^24^, ^25^ All of these elements mean that a certain routine, getting to know the patient during the years, may result in an unintentional but noticeable omission, noticed by patients.

The therapeutic relationship between the nurse and the patient, in the opinion of the respondents, is of great importance in renal replacement therapy, but only 87% of them considered the nurse's current relationship with them to be therapeutic. Despite such a good assessment, patients expected an increase in the involvement of nursing staff in the therapeutic relationship, which should be noted, taking into account socio‐demographic variables, the duration of therapy, and its acceptance by the patient or lack thereof.

The study was limited due to a small number of respondents and due to the involvement of only a single dialysis center. More research is needed on a larger group of patients with the use of standardized tools.

### 4.1 Implications for clinical practice

The work of a nurse is characterized by contact with another person that is sick or at risk of a disease and often undergoes a specific therapy. Therefore, the therapeutic relationship with the patient should be the basis of every nurse's work. The establishment of a therapeutic relationship that is experienced by the patient as safe, supportive, and trustworthy is not a simple thing, but very important, as an expression of the nurses' commitment and profound professional responsibility. The hemodialysis procedure is a repeated procedure, lasting about 4 h, which the nurse can use to support the patient by respecting the patient, genuinely interested in him as a person, emotional warmth, tolerance, and nonjudgmental acceptance of the patient, especially the ability to listen carefully, empathy, and real patient's own strength and abilities, while being aware of the limitations resulting from the disease or therapy, and presenting appropriate health behaviors. Emphasis should be placed on the individual character of the therapeutic relationship, the need to recognize the needs of a specific individual, and, based on them, implement a specific action plan. It seems necessary to pay more attention to establishing and maintaining a therapeutic relationship, the advantages of which are noticed by patients. It also influences the broadly understood quality of care.

## 5 CONCLUSIONS

The concept of a therapeutic relationship and its elements are known to the respondents. Its correctness has a positive effect on all spheres of the bio‐psycho‐social condition of patients, stimulating them to cooperate in the fight against the disease and in therapy. The establishment of a therapeutic relationship with the patient should be the basic course of action of every nurse. Nursing care in conjunction with the therapeutic relationship achieves the right quality in the care of a hemodialysis patient.

## CONFLICT OF INTERESTS

The authors declare that there are no conflict of interests.

## References

[1] O'Brien AJ. The therapeutic relationship: historical development and contemporary significance. J Psychiatr Ment Health Nurs. 2001;8(2):129‐137. 11882118 10.1046/j.1365-2850.2001.00367.x

[2] Kornhaber R, Walsh K, Duff J, Walker K. Enhancing adult therapeutic interpersonal relationships in the acute health care setting: an integrative review. J Multidiscip Healthc. 2016;9:537‐546. 27789958 10.2147/JMDH.S116957 PMC5072574

[3] Feo R, Conroy T, Wiechula R, Rasmussen P, Kitson A. Instruments measuring behavioural aspects of the nurse‐patient relationship: A scoping review. J Clin Nurs. 2020;29(11‐12):1808‐1821. 31162861 10.1111/jocn.14947

[4] Bielawska J Interdisciplinary character of nursing. Zeszyty Naukowe Państwowej Wyższej Szkoły Zawodowej im. Witelona w Legnicy. 2014;11(2):9‐20.

[5] Del Piccolo L, Goss C. People‐centered care: new research needs and methods in doctor‐patient communication. Challenges in mental health. Epidemiol Psychiate. 2012;21:145‐149. 10.1017/S2045796012000091 22789161

[6] Williamsa J, Stickley T. Empathy and nurse education. Nurse Educ Today. 2010;30:752‐755. 20381220 10.1016/j.nedt.2010.01.018

[7] Falter B. Relationship‐based care: a model for transforming practice. Nurs Admin Q. 2006;30(2):182‐190.

[8] United States Renal Data System 2018 USRDS annual data report Epidemiology of kidney disease in the United States. National Institutes of Health, National Institute of Diabetes and Digestive and Kidney Diseases, Bethesda, MD, 2028. https://www.uslegalforms.com/jsfiller-desk13/?requestHash=a48f0241330255d64741df5b860d94f05f59303d2a23d8dfb3baf99ad2887a82%26ref=https://www.uslegalforms.com%26projectId=679582105%26et=as#b43f35b17628712b54f8a55d27587c44. Accessed March 3, 2021.

[9] Himmelfarb J, Vanholder R, Mehrotra R, Tonelli M. The current and future landscape of dialysis. Nat Rev Nephrol. 2020;16:573‐585. 32733095 10.1038/s41581-020-0315-4 PMC7391926

[10] Kalinowska A, Kowalczyk M, Pruszko C, Prystacki T. The current state of dialysis in Poland – 2018 y. Report. Neprol Dial Pol. 2019;23:3‐4.

[11] Kopański Z, Wojciechowska M, Antos E, Uracz W, Beczek M. The significance of interpersonal communication in nursing. J Public Health Nurs Med Rescue. 2013;3:28‐31.

[12] Malou MPG, Van der Heijden KB, Huijbregts SCJ, Van Goozen SHM, Swaab H. Associations between empathy, inhibitory control, and physical aggression in toddlerhood. Dev Psychobiol. 2019;62:871‐881. 10.1002/dev.21951 PMC7496157 31998974

[13] Motyka M. The role of active listening in therapeutic communication with patient. Nursing Problems. 2011;19(2):259‐265.

[14] Mularska‐ Kucharek M. Trust – A fundamental component of social life. A study of the Lodz Voivodeship. Studia Regionalne i Lokalne. 2011;2(44):76‐91.

[15] Nowakowska E. Morphology of relationship bondings in healthcare institutions. Wydawnictwo Naukowe Uniwersytetu Szczecińskiego. 2015;39:285‐297.

[16] Radecka I, Łopacińska I, Kopański Z. The significance of interpersonal communication in nursing. Journal of Clinical Healthcare. 2014;4:6‐7.

[17] Report of the Supreme Council of Nurses and Midwives 2017. Warsaw, Poland: Main Chamber of Nurses and Midwives; 2017. https://nipip.pl/wp-content/uploads/2017/03/Raport_druk_2017.pdf Accessed October 27, 2020.

[18] Shukla RK. Technical and structural support systems and nurse utilization: systems model. Inquiry. 1983;20:381‐389. 6229489

[19] Skuza B, Włodarczyk D In: Jakubowska‐Winecka A, Włodarczyk D (ed.), Znaczenie relacji pacjent‐personel medyczny dla przebiegu leczenia [In:] Psychologia w praktyce medycznej. Warszawa: PZWL; 2010:134‐135.

[20] Welch M. Pivotal moments in the therapeutic relationship. J Psychiatr Ment Health Nurs. 2005;14(3):161‐165. 10.1111/j.1440-0979.2005.00376.x 16181152

[21] Sowińska K, Kretowicz K, Gaworska‐Krzemińska A, Świetlik D. The burnout syndrome and job satisfaction in the opinion of nurses. Nursing Problems. 2012;20(3):361‐368.

[22] McCabe C. Nurse–patient communication: an exploration of patients' experiences. J Clin Nurs. 2004;13:41‐49. 14687292 10.1111/j.1365-2702.2004.00817.x

[23] Woynarowska B. Edukacja w grupach ryzyka [in:] Edukacja zdrowotna. Wydawnictwo Naukowe PWN, Warszawa. 2017:448‐449.

[24] Zarzycka D, Ślusarska B, Dobrowolska B, Deluga A, Trojanowska A, Bartoń E. Empathy in nursing. Assumptions, practice and its empirical determinants. Nursing in the 21st Century. 2016;3:34‐35.

[25] Merkes M. Mindfulness‐based stress reduction for people with chronic diseases. [in:] Database of Abstracts of Reviews of Effects (DARE): Quality‐assessed Reviews [Internet]. York (UK): Centre for Reviews and Dissemination (UK); 2010. https://www.ncbi.nlm.nih.gov/books/NBK80061/. Access 27.10.2020.

